# The Influence of English as a Foreign Language Teachers’ Positive Mood and Hope on Their Academic Buoyancy: A Theoretical Review

**DOI:** 10.3389/fpsyg.2021.801435

**Published:** 2022-01-04

**Authors:** Hui Dong, Wei Li, Di Ye

**Affiliations:** School of Foreign Studies, Tangshan Normal University, Tangshan, China

**Keywords:** academic buoyancy, emotions, positive psychology, positive mood, hope

## Abstract

Emotions are now considered critical elements of a successful education. In English as a Foreign Language (EFL) context, there are many challenges for teachers to deal with. Hence, it is necessary to take their emotions into consideration. Despite many studies in this area, researching teachers’ positive mood, hope, and academic buoyancy has been left less attended. Trying to introduce this line, the present study reviewed the definitions, related concepts, theories, and previous studies done on these three variables in detail. It also touched upon the origins of researching emotion in educational contexts describing different schools of psychology. Additionally, the study offered some practical implications for EFL teachers, students, policy-makers, teacher trainers, and researchers. Finally, it enumerated the existing gaps in this area and made a number of research suggestions for future research.

## Introduction

It is now a common belief among many scholars and educators that emotions and feelings have a quintessential role in language teaching and learning (Mercer, 2020). Such inner states can permeate into every single aspect of one’s career including his/her performance, behavior, and instructional practices (Sikma, 2021). This shift of attention to psycho-emotional factors in education took root from the humanistic psychology of the time and a new trend known as positive psychology (PP), which places one’s positive emotions at the highest priority (Gregersen and MacIntyre, 2021). It accentuates the criticality and rigor of positive feelings (e.g., joy, credibility, love, engagement, rapport, immediacy, passion, resilience, clarity, optimism, care, etc.) in driving one forward in his/her profession and generating numerous optimal outcomes (Zhang and Zhang, 2020; Wang and Derakhshan, 2021; Wang et al., 2021). In teaching a second/foreign language, the importance of affect and inner states multiply due to the challenging and stressful nature of this job (Benevene et al., 2020). Other than pedagogical demands, an EFL/ESL teacher must be able to cope with myriads of cultural, linguistic, ideological, and socio-political disparities and setbacks (King and Ng, 2018). This stability and toughness of teachers, as architects of societies, requires a dramatic transition from negative stressors (e.g., anxiety, tension, fear, burnout etc.) to positive constructs in teacher psychology (Jin et al., 2020; Pishghadam et al., 2021b).

Reviewing the pertinent literature in this domain, one can notice that different investigations have been carried out in different contexts certifying the power of teacher’s positive emotions in bringing about many desirable outcomes in academia such as increased self-efficacy, self-esteem, motivation, job satisfaction, resilience, credibility, immediacy, achievement, interpersonal communication skills, work engagement, professional identity, well-being, agency, and many more (e.g., Derakhshan et al., 2019, 2020; Jin et al., 2020; Li and Yang, 2021; Pishghadam et al., 2021a; Sun, 2021; Xie and Derakhshan, 2021; Zhao and Li, 2021, among others). These outcomes are achievable only in a positive, caring, and democratic educational culture where forming a positive mood in the teachers is the first stepping stone. When teachers have a positive mood, they are more focused, flexible, creative, confident, and optimistic about their job (Febrilia and Warokka, 2014). Consequently, they put their heart and soul more into their practices to help students learn better. Although positive mood seems to be a transient and context-specific construct in teachers’ pedagogy, it can reach a stable stage as teachers gain more experience (Newton, 2013). A durable positive mood can be claimed to facilitate and reinforce the construction of other critical positive emotions in teachers like “hope” which are both less explored in positive psychology research. By definition, hope refers to a positive mental state in which the individual specifies clear goals for him/herself, maintains his/her determination, and tries alternative paths to defeat setbacks and lead to a positive future (Snyder et al., 1991; Ghadyani et al., 2020). Research has indicated that teachers’ hope has a direct relationship with their mood and influences their well-being, optimism, self-efficacy, satisfaction; success, and academic performance (Alarcon et al., 2013; Ong et al., 2018).

In the context of L2 education, which is full of adversities, challenges, and inconsistencies, EFL teachers usually have to grapple with numerous socio-economic agonies and despondencies which are continuously slowing their training ship (Ghadyani et al., 2020). This gives more prominence to the criticality of hope in language education in order to stay positive, look on the bright side, and be tough in the face of challenges inborn in L2 education. Without hope, EFL teachers cannot pass through academic difficulties or be academically buoyant; a new concept in teacher psychology defined as one’s capability to handle, endure, and overcome the existing setbacks and adversities of his/her educational career (Comerford et al., 2015). Buoyancy like many other psychological factors can be affected by inner and outer factors. In other words, both internal feelings and contextual variables have a role to play in a teacher’s level of buoyancy (Zhang, 2021). Despite the existence of empirical studies on the construct of academic buoyancy, as a positive form of resilience, they have been limited to EFL students (e.g., Putwain et al., 2015; Yun et al., 2018) and few studies have explored this variable in relation to EFL teachers, its impact on their instruction, and whether it can be predicted by other teacher emotions or not (Zhang, 2021). To fill this gap, the present review article was an effort to review the definitions, theoretical underpinnings, empirical findings, and associations between three less explored variables in teacher psychology, namely positive mood, hope, and academic buoyancy.

## Background

### Emotions in Education: Theories and Origins

For a long time, learning and teaching processes were carried out by practitioners without taking into account the role of psycho-emotional factors. Opposing this view, a number of breakthroughs in psychology and pedagogy emerged to tackle the limited outlook of constructivism that ignored the role of emotions in one’s development (Li, 2021). The current centrality of emotions in education and language education emanated from the propositions of humanistic psychology, positive psychology, positive peace psychology, and a broad paradigm called affective turn in pedagogy. These giant steps grew out of each other having many things in common. More specifically, positive psychology (PP, hereafter) was an extension of humanism but focused on the power of positive emotions in causing happiness and development in life (MacIntyre et al., 2019). In contrast to traditional views, which lingered on negative sides and factors in learning, PP put emphasis on the positive sides of life, while approved and considered the dire consequences of adversities and problems (Seligman, 2011; Zhang and Zhang, 2020). As stated by Seligman (2011) and MacIntyre and Mercer (2014), PP draws on three rigorous pillars including *positive subjective experience* (or PSE, emotions), *positive individual traits* (or PIT, individual characteristics), and *positive institutions* (or PI, contexts). Based on these cores, one can thrive and reach a high performance as long as his/he inner states, personality features, and contextual factors allow him/her to do so. Instead of abnormalities, PP explores how resilience, optimism, engagement, mood, outlook, joy, love, hope, passion, positive interpersonal skills, and enjoyment can drive one’s performance forward (Dewaele et al., 2019; Wang and Derakhshan, 2021).

PPP, on the other hand, goes beyond PP and highlights the role of positive interpersonal relations, peace, and classroom rapport in developing students’ academic gain (Gibson, 2011). Akin to PP, PPP examines the influence of positive emotions on peace-building at various levels which bring other optimum outcomes as well. Both schools of psychology mention, were inspired by a larger tradition recognized as “*affective pedagogy”* (AP) that is a way of instruction whose aim is to arouse particular emotional states (Ainsworth and Bell, 2020). AP itself built upon the ideas of *the affective turn* (Clough, 2007) that highlighted emotional experiences and feelings in learning process. In other words, AP focuses on teachers’ psycho-pedagogy in order to develop students’ affects/emotions required for success (Williamson, 2016).

The result of this concentration on emotions in education has led to numerous empirical and conceptual studies on various positive emotions (constructs) including self-efficacy, self-esteem, resilience, immunity, stroke, motivation, engagement, love, credibility, clarity, enjoyment, praise, immediacy, hope and the like which were approved to affect teachers’ and students’ behaviors and practices (e.g., Dewaele et al., 2019; Fathi et al., 2020; Zhang and Zhang, 2020; Pishghadam et al., 2021a; Wang et al., 2021; Xie and Derakhshan, 2021; Zhao and Li, 2021, to cite a few). However, there are still many other positive emotions to be explored in L2 education, especially in relation to EFL teachers.

### The Definition of Positive Mood

The concept of positive mood has been limitedly explored in teacher/learner psychology leading to only a couple of definitions. This is mainly due to the existing cognate terms in this area whose demarcation boundaries are not clear. To reach a solid description for the construct of positive mood, one needs to distinguish among “affect,” “emotion,” and “mood.” Although they seem equal, they have minor disparities as well. Affect is an overarching term covering both emotions and moods. Affect is one’s basic feeling (either pleasant or unpleasant), while emotion is a more complicated mental state with clear causes/antecedents ending in reactions. It is temporary but can lead to mood, which refers to relatively continuous affective states happening without any reason/cause (Bryan et al., 1996). Moreover, moods have low intensity but are self-regulated to offer information about one’s state (Newton, 2013). Drawing on these explanations and differentiation, positive mood can be defined as a momentary mental state emerging when one has confidence, optimism, flexibility, and positive evaluation of something (Park, 2002). In language education, when a teacher or a student forms a positive mood, he/she feels good about his/her actions and invests more time and energy to incur positive outcomes and achievements.

### The Potentialities of Positive Mood in Education

Now that the pivotal role of emotions in education has been strongly substantiated by growing bodies of research, it is evident that having a positive mood toward something in academia brings about many other favorable results like a chain of rings. Mood is a general state showing one’s feeling, confidence, and enjoyment of doing an academic task/practice. Hence, being positive and feeling positive in education influence teachers’ and students’ various behaviors and practices in the classroom. In brief, research shows that a positive mood can have a considerable impact on one’s confidence level, optimism, resilience, hope, outlook, cognition, efficacy, creative thinking, information processing ability, problem-solving, productive thinking, task management, work/classroom engagement, interpersonal communication/interaction abilities, cognitive control, flexibility, and decision-making). Undoubtedly, many other constructs related to learner and teacher psychology can also be added to this list suggesting the power of positive mood in education and its increasing potentials for different stakeholders.

### The Notion of Hope

The notion of hope is one of the most important constructs in positive psychology, yet it has received scant attention among scholars. Hence, the theoretical and operational definitions for the concept of hope are not many. However, in their seminal work, Snyder et al. (1991) conceptualized it as the process in which an individual thinks about his/her goals with motivation to pursue those goals using different ways to obtain those goals. Based on this definition, hope is way beyond a simple emotion and it is a dynamic, cognitive, motivational process with three components of “goal,” “agency,” “and “pathways” by which the person sets clear goals, maintains his/her will/determination, and takes advantage of different tools and strategies to deal with difficulties in meeting those goals (Snyder, 2002). In other words, hope is a motivational inner feeling emerging out of the mentioned three elements which work additively and reciprocally. Therefore, it is a perceived capacity to derive pathways to favorite goals, and encourage oneself through agency thinking to employ those pathways (Rand and Cheavens, 2009).

### Theories Behind Hope: Hope Theory and Self-Determination Theory

The most notable theory behind the concept of hope was proposed by Snyder, an American positive psychologist, who explored the connection between one’s hope and his/her performance and well-being. Snyder’s hope theory encompassed three components of goals, pathways, and freedom of choice (agency). He considered goals as the central tenet of hope theory since much of human behavior is purported to be goal-oriented (Snyder, 1998). In this theory, goals are verbal or visual depictions of the targets that direct one’s action. Later, Snyder (2002) divided human goals into “approach goals” (i.e., positive targets you want to achieve) and “avoidance goals” (negative things you want to avoid). According to this theory, pathways refer to one’s perception of his/her ability to create alternative routes toward their imagined future targets. This is significant when people face challenges and setbacks in the course of achieving their goals. The final core of hope theory concerns agency or the ability to keep and continue one’s motivation and attempt when using a pathway. It is similar to the concept of self-efficacy in that they reflect one’s perception and ability to do something efficiently. However, self-efficacy is a perceived ability, while agency is a global and definite ability to do a task. In simple words, self-efficacy is perception-oriented, while agency is intention-oriented (Rand and Cheavens, 2009).

Another theoretical basis of hope is related to self-determination theory (SDT) proposed by Deci and Ryan (1985). SDT is macro-theory of motivation, which draws on the ideas of agentic behavior in human beings (Adams et al., 2017). The theory rests on three elements of ***competence*** (one’s desire to master the environment and experience satisfaction), ***autonomy*** (one’s freedom of choice and volition in doing an action and being the origin of his/her action), and ***relatedness*** (a sense of social belongingness to others) as three psychological needs to be satisfied. SDT is related to the construct of hope in that it argues for agentic actions and causal agency by detecting pathways that help meeting one’s desires and getting involved in self-direction and self-regulation of his/her actions to better cope with challenges and opportunities (Marques and Lopez, 2018). Hence, it explicitly reflects the three components of hope (goals, agency, and pathways).

### Hope and Language Education: Correlates and Outcomes

Tracing the pertinent literature on the concept of hope, one can identify various outcomes and variables related to hope in educational settings. As research indicates, hope has a correlation with and predicts academic achievement, academic performance, resilience, confidence, self-esteem, career behavior, success, satisfaction, cognitive ability, self-efficacy, psychological well-being, and engagement (Hirschi, 2014; Feldman and Kubota, 2015; Gallagher et al., 2017). Despite these studies that highlighted the role of hope, few studies (if any) have specifically examined hope in the context of language education and L2 learning and teaching. Moreover, the construct of hope has not been operationally defined and statistically measured in SLA (Ghadyani et al., 2020) leaving many rooms open for future research on other positive psychology variables to see if hope can predict additional SLA variables or not.

### Academic Buoyancy: Definitions and Related Terms

Academic buoyancy is one of the novel variables of PP, which highlighted the influence of emotions in learning and teaching. Simply, it pertains to one’s ability to identify and deal with academic adversities and setbacks that appear in one’s academic life (Martin and Marsh, 2019). In contrast to resilience that focuses on general difficulties, academic buoyancy is a psychological variable that centers on daily academic setbacks (Yun et al., 2018; Jahedizadeh et al., 2019). Hence, it is a sort of positive, constructive, and adaptive response to existing academic challenges (Putwain et al., 2012). Like many other PP constructs, academic buoyancy is affected by internal (personality traits and variables) and external factors (contextual issues) (Comerford et al., 2015). Additionally, it capitalizes more on strengths than weaknesses, takes proactive and not reactive approaches in the face of challenges, and examines “many and healthy” cases instead of “extreme cases” (Martin and Marsh, 2019). Owing to this positive emotion orientation, academic buoyancy is known as the positive form of resilience (Zhang, 2021).

In the relevant literature, different cognate terms have been proposed and used in association with/place of academic buoyancy including *resilience*, *coping, immunity*, and *hardiness*. Although they seem equal and with overlaps, they vary along some lines. For example, resilience does not explain the challenges that regularly occur in academic contexts since it solely focuses on a small and extreme group of cases (Phan and Ngu, 2014; Martin and Marsh, 2019). Furthermore, it varies from academic buoyancy in theoretical and operational definition, sampling, population, measurement, and methodology (Jahedizadeh et al., 2019). Another similar concept is called coping which refers to one’s strategies to solve the problems or modify the way they are perceived (Somerfield and McCrae, 2000). They are specific techniques used to deal with an aversive issue. Immunity is the third cognate term that is taken from the biology and medical science lexicon to point to the defensive mechanisms that an individual employs to reduce and prevent the challenges, turbulences, and harms to his/her motivation, behaviors, professional identity, and practice (Hiver, 2017). The distinctive feature of this concept comparing to resilience and academic buoyancy is that immunity is spontaneous and double-edged at the same time in the sense that it can be both productive and counterproductive depending on its use, manner, and timing (Hiver, 2017). The last similar concept here is hardiness that concerns one’s capacity to combat and diminish the negative effects of stress on one’s performance and practice (Hiver and Dörnyei, 2017).

### Academic Buoyancy and SLA

It is over a decade that the concept of academic buoyancy has entered into the realm of education and research with the groundbreaking work of Martin and Marsh (2008). The construct gained a growing bulk of research due to the overwhelming adversities and challenges inborn in L2 education. Hence, to perform satisfactorily in EFL contexts, one needs to know the difficulties and prepare proper coping strategies not to fringe in the face of adversity. With this knowledge, over the past years, different scholars from different parts of the world have conducted investigations on buoyancy signifying its positive effects on students’ achievement, motivation, participation, self-efficacy, persistence, self-esteem, exam performance, sustainability, competence, classroom enjoyment, self-regulation strategies, engagement, commitment, and decreasing stress and anxiety (e.g., Malmberg et al., 2013; Yun et al., 2018; Jahedizadeh et al., 2019; Wang and Guan, 2020; Zhang, 2021). Despite these studies and the motivational cores unpacked by this concept, it has been mostly explored from the vantage point of students and investigations in EFL/ESL contexts are still insufficient in this area. Consequently, academic buoyancy calls for more research in SLA to provide operational definitions, models, frameworks, and methodological tools to disclose the challenges of SLA and their suitable coping strategies in buoyancy.

### Implications, Research Gaps, and Future Directions

In this study, it was argued that EFL teachers’ positive mood and hope can predict and improve their level of academic buoyancy to stand and tackle the existing challenges and setbacks of learning and teaching a second/foreign language. Hence, this review article can be beneficial for EFL teachers in that they can raise their understanding about the power of having a positive mood and hope in their profession to improve and generate many desirable outcomes in learning and teaching, especially in the face of adversities. They can also use the ideas raised in this review article to devise and use appropriate strategies to establish positive moods, stay hopeful, and be tough in dealing with the challenges of L2. Likewise, the study would be of help for EFL students in the sense that they can realize their role in teachers’ mood and level of hope. Hence, behave and work in a way that these constructs are promoted in their teachers, which, in turn, contribute to various positive outcomes in students as well. Furthermore, EFL teacher trainers can use this article as a starting point for running professional development courses for novice EFL teachers concerning the emotional sides of teaching along with pedagogical issues. Similarly, the ELT community can benefit from this review study in that scientific and professional conferences and knowledge-development programs can be designed where the inherent adversities and setbacks of L2 education and the ways to eradicate them are fully discussed. Additionally, such programs can increase the awareness of the overall community about the role of positive emotions and hope in language education.

Furthermore, policy-makers and curriculum designers can benefit from this study in that they can revisit their stance on the role of emotions in teaching and make efforts to reinforce teachers’ positivity, hope, and buoyancy in a profession known as one of the most challenging jobs. Finally, L2 researchers can take advantage of this theoretical analysis and run similar studies on teachers’ mod, hope, and buoyancy. As reviewed, many existing studies in this domain are correlational and one-shot, hence experimental and longitudinal studies are recommended. Likewise, this line of research has mostly been scrutinized from the perspective of students, while teachers’ stance can add more to the concept. Moreover, these variables are dynamic and context-sensitive and these features inspire running cross-cultural and time-series studies to see if various cultures have different views about these factors and propose additional components for them or not. Additionally, passionate researchers can replicate this study using mixed-methods designs and qualitative tools to capture the dynamics and developmental paths of teachers’ positive mood, hope, and academic buoyancy. Similarly, many studies in this area have been conducted in English-speaking countries where challenges in academia may be less that in EFL contexts. So, running empirical investigations in EFL settings is suggested. Despite the insightful findings gained through previous studies in this area, it is evident that there are still many uncharted roads for research left for keen scholars.

## Author Contributions

All authors listed have made a substantial, direct, and intellectual contribution to the work, and approved it for publication.

## Conflict of Interest

The authors declare that the research was conducted in the absence of any commercial or financial relationships that could be construed as a potential conflict of interest.

## Publisher’s Note

All claims expressed in this article are solely those of the authors and do not necessarily represent those of their affiliated organizations, or those of the publisher, the editors and the reviewers. Any product that may be evaluated in this article, or claim that may be made by its manufacturer, is not guaranteed or endorsed by the publisher.
